# Lumbar position sense acuity during an electrical shock stressor

**DOI:** 10.1186/1471-2474-6-37

**Published:** 2005-07-01

**Authors:** Nis Hjortskov, Christian Hye-Knudsen, Nils Fallentin

**Affiliations:** 1 Department of Physiology, National Institute of Occupational Health, Lersø Parkallé 105, DK 2100 Copenhagen, Denmark

## Abstract

**Background:**

Optimal motor control of the spine depends on proprioceptive input as a prerequisite for co-ordination and the stability of the spine. Muscle spindles are known to play an important role in proprioception. Animal experiments suggest that an increase in sympathetic outflow can depress muscle spindle sensitivity. As the muscle spindle may be influenced by sympathetic modulation, we hypothesized that a state of high sympathetic activity as during mental stress would affect the proprioceptive output from the muscle spindles in the back muscles leading to alterations in proprioception and position sense acuity. The aim was to investigate the effect of mental stress, in this study the response to an electrical shock stressor, on position sense acuity in the rotational axis of the lumbar spine.

**Methods:**

Passive and active position sense acuity in the rotational plane of the lumbar spine was investigated in the presence and absence of an electrical shock stressor in 14 healthy participants. An electrical shock-threat stressor lasting for approximately 12 minutes was used as imposed stressor to build up a strong anticipatory arousal: The participants were told that they were going to receive 8 painful electrical shocks however the participants never received the shocks. To quantify the level of physiological arousal and the level of sympathetic outflow continuous beat-to-beat changes in heart rate (beats*min^-1^) and systolic, diastolic and mean arterial blood pressure (mmHg) were measured. To quantify position sense acuity absolute error (AE) expressed in degrees was measured. Two-way analysis of variance with repeated measurements (subjects as random factor and treatments as fixed factors) was used to compare the different treatments.

**Results:**

Significant increases were observed in systolic blood pressure, diastolic blood pressure, and heart rate during the stress sessions indicating elevated sympathetic activity (15, 14 and 10%, respectively). Despite pronounced changes in the sympathetic activity and subjective experiences of stress no changes were found in position sense acuity in the rotational plane of the lumbar spine in the presence of the electrical shock stressor compared to the control period.

**Conclusion:**

The present findings indicate that position sense acuity in the rotational plane of the spine was unaffected by the electrical shock stressor.

## Background

Epidemiological studies have identified associations between work related psychosocial stressors and low back disorders [1,2]. The physiological mechanisms and pathways linking work stressors to low back disorders are uncertain.

In laboratory studies arousal or mental stress impaired the performance of different motor tasks. An electrical shock stressor was associated with reductions in steadiness of a pinch grip task [3,4] and the presence of mental stressors using non-supportive language and actions increased the spine compression during a standardized lifting task [5,6].

Optimal motor control of the spine depends on proprioceptive information as a prerequisite for coordination and the stability of the spine [7]. Position sense, functionally defined as the awareness of the actual position or movement of the limb, in the lumbar spine is influenced by low back pain [8-10], muscle fatigue [11] and muscle vibration [12]. The muscle spindle afferents play a major role in the sensation of position and movement [13-15] and factors altering the muscle spindle sensitivity may affect the proprioception.

Animal studies have shown the presence of sympathetic fibres [16] and adrenergic receptors inside the muscle spindle [17], and demonstrated how sympathetic stimulation during a muscle stretch reduced the muscle spindle sensitivity in rabbit jaw muscles [18,19]. In studies of human muscles, no detectable change in resting discharge of spindle firing during a period of increased muscle sympathetic activity (MSNA) was found [20]. Further, Matre & Knardahl [21] demonstrated that proprioceptive acuity was unchanged or, in one condition, improved during muscle sympathetic activation. Whether and how an increased sympathetic activation of the muscles spindles affect the proprioceptive acuity in human muscles are thus far from evident, and to the authors knowledge no studies have investigated the effect of mental stress, i.e. the response to an electrical shock stressor, on proprioceptive acuity in the low back region. The diversity of sympathetic outflow to different muscle groups during mental stress, i.e. mental stress increased MSNA in the calf but not in the forearm [22], should also be mentioned. In this regard it is unknown whether mental stress increases MSNA in the back muscles. It has however been suggested that when motor tasks requiring precision and continuous proprioceptive feedback are performed in work situations with strong excitement and stress, the enhanced muscle sympathetic outflow may affect motor performance through the muscle spindle system [18]. We examined whether a state of high sympathetic activity would affect the proprioceptive acuity of the back muscles. Therefore, the aim was to investigate the effect of mental stress, in this study the response to an electrical shock stressor, on position sense acuity in the rotational axis of the lumbar spine.

## Methods

### Participants

Fourteen healthy participants, 8 female and 6 male students (age 23.4 (SD 1.3) years, body mass 66.8 (SD 9.9) kg and height 173 (SD 9.5) cm) participated in the study. The participants had no history of injury or current problems with the low back. The local ethics committee of Copenhagen approved the study. All participants gave their informed consent.

### Procedure

**Figure 1:**
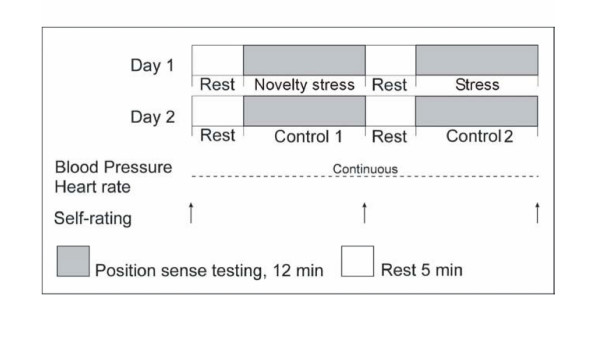
**Procedure. **The experimental protocol at day one and day two. Arrows indicate the time the participants reported their subjective experiences of stress. "Stress" refers to the exposure to the electrical shock stressor.

The procedure is illustrated in Figure 1. The study was performed over two days (2–4 days in between). The participants performed two position sense tasks (i.e. a passive and an active position sense task. These are described in the "position sense task section" below) during each of the following periods: a "stress period", a "novelty stress period" (day 1) and during two control periods (day 2).

The participants were exposed to an electrical shock stressor in the "stress period", i.e. the participants were told that they would receive 8 painful electrical shocks in this period (for further description of the stressor, see the electrical shock stressor section). The "novelty stress period" was without the exposure to the electrical shock stressor, but being anxious/nervous for participating in the experiment resulting in markedly elevations in the physiological stress markers (blood pressure and heart rate) and anxious/nervous for the stressor in the following "stress period". The "novelty stress", "stress" and the two control periods lasted 12 minutes each. Five minutes rest separated the "novelty stress" and the "stress" periods on day one and the control 1 and control 2 periods on day two. Prior to the testing periods the participants rested in 5-minutes. The procedure was the same on day two (control day) except that the "novelty stress" and the "stress" periods were changed to control periods (control 1 and 2).

It was not possible to randomise the order of the stress and control periods because pilot tests indicated that the physiological markers of stress (blood pressure and heart rate) were permanently elevated on day one due to novelty with the laboratory surroundings, and did not return to resting levels when the control and stress periods were performed in one day. This is also confirmed by the fact that there are order effects in resting blood pressure, i.e. the blood pressure was even increased in the resting period between the "novelty stress" and the "stress" periods. Lack of randomisation is in accordance with previous studies investigating the effect of mental stressors on motor performance [3-5,23].

### Position sense tasks

Subjects were seated in a car-like chair of the Biodex System III Isokinetic Dynamometer (Biodex Medical Inc., Shirley, NY, USA) in a motorised rotational back attachment. The upper part of the trunk was strapped to the attachment with a belt at the level of the deltoid muscle, and the thighs were strapped to the chair. Further, the arms were strapped to the attachment in front of the participant. Horizontal rotations in the rotational plane of the lumbar spine from right to the left were performed from a starting position of 0° (relative to the sagittal plane) to target positions of 10°, 20° and 30° in a range of motion from 0° to 40°. The dynamometer was locked in the 0° position to ensure the same starting position in all the tests. Four trials were performed for each target position in each position sense task i.e. the participants performed 12 target positions in each position sense task. The order of the target positions and the passive and active tasks was randomised.

In the passive task, the trunk was passively moved at an angular velocity of 10°s^-1 ^to a pre-determined target position. The target position was unknown to the participant to avoid the participants to predict the target position. The experimenter stopped the dynamometer at the target positions. The rotational attachment was then locked and the trunk remained at the target position for 5 s. Then the participant was passively returned at 10°s^-1 ^to the starting position. After remaining in the starting position for 5 s the trunk was passively moved at 10°s^-1 ^and stopped when the participant pressed a trigger. The trigger indicated recognition of the target position. In the active task the participant actively moved the trunk from the starting position until a command to stop was given. The rotational attachment was locked and the trunk remained at the target position for 5 s. Then the participant actively moved the trunk to the starting position remaining there for 5 s. The participant actively moved the trunk to match the target position and they indicated when the trunk was considered to be at the target position.

The test-retest reliability (2 days in between test and retest) of the passive and active position sense procedures was tested in a pilot study involving 10 participants. The statistical analysis showed no difference between the test and retest for the passive and active tasks. Based on the results of the intra class correlation (ICC as an estimate of reliability) (0.46 for the active task and 0.69 for the passive) and standard error of measurement (SEM as an estimate of precision) (0.64° in the active task and 0.39° in the passive task) values, the test reliability and precision for spinal position sense testing were moderate according to the criteria of Shrout & Fleiss [24]. These values are in accordance with previous studies e.g. [25-27]. The relatively low SEM values expressed in absolute error in the passive and active procedure indicate relatively good and precise test stability [28].

Sources of errors were minimized by using: the same experimenter in all trials; standardised verbal instructions, i.e. the participants received identical instructions about the proprioceptive tasks in the novelty stress, stress and control conditions; blindfolding to eliminate visual cues; randomisation of target positions; to keep each trial short (approx. 45 min), and familiarization with the principles in the testing procedure.

### Measurements

The participants reported subjective experiences of stress prior to testing and after each of the periods at day one and two. The following four 11-point scales (0 = not at all, 10 = extremely) were used: 1) stressed, 2) tensed, 3) exhausted and, 4) concentrated [29,30].

To quantify the level of physiological arousal, non-invasive continuous beat-to-beat changes in heart rate (HR) (beats*min^-1^) and systolic (SBP), diastolic (DBP) and mean arterial blood pressure (MAP) (mmHg) were measured with an inflatable cuff placed over the proximal portion of the middle finger connected to a Finometer™ device (Finapres Medical Systems BV – TNO TPD Biomedical Instrumentation, The Netherlands) and recorded in a computer. The heart rate and blood pressure data were analysed using BeatScope software package version 1.1 (TNO TPD Biomedical Instrumentation, The Netherlands). The blood pressure was automatically corrected for hydrostatic pressure to compensate for vertical movements of the hand with respect to heart level and the concomitant pressure changes in the finger blood pressure.

The angular positions were recorded in a computer using a LabView program (National Instruments). The absolute errors (AE), i.e. the absolute values of the difference between the reproduced position and the target position, were determined using a MatLab programme. For testing differences in distance the mean AE for each target position (10°, 20° and 30°) for each subject was computed, and for testing differences in procedures (passive and active) and periods (novelty stress, stress and control 1 and control 2) the mean AE for each procedure and each period were computed.

### Electrical shock-threat stressor

Prior to the stress period two circular dummy "shock electrodes" were attached to the dorsal side of the right forearm. The electrodes were attached to an electrical stimulation device. The aim was to build up a strong anticipatory arousal thereby strongly activating the sympathetic nervous system [31]. During the stress period – the participants did not receive any electrical shocks in the experiment – the participants were told that they were going to receive 8 painful electrical shocks without any presage either during the passive or active position sense task. To further heighten the arousal, the participants were told that they would receive additional electrical shocks every time the absolute error was exceeding 5°. When the first part of the stress period was terminated without any electrical shocks the participants were assured that they would receive the electrical shocks in the subsequent part of the stress period.

### Statistics

Two-way analysis of variance with repeated measurements (subjects as random factor and treatments i.e. the novelty stress, stress and control 1 and 2 as fixed factors) was used to compare the different treatments. If significant differences appeared, multiple comparisons (Tukey test) were used to isolate the treatment that differed from the others. Mean AE for each subject for each target position was calculated. Mean AE, SBP, DBP, MAP and HR were dependent variables. Furthermore, median values of self-reports on stress, tenseness, exhaustion and concentration were dependent variables. Level of significance was set to P < 0.05.

## Results

### Subjective experience of stress

**Figure 2:**
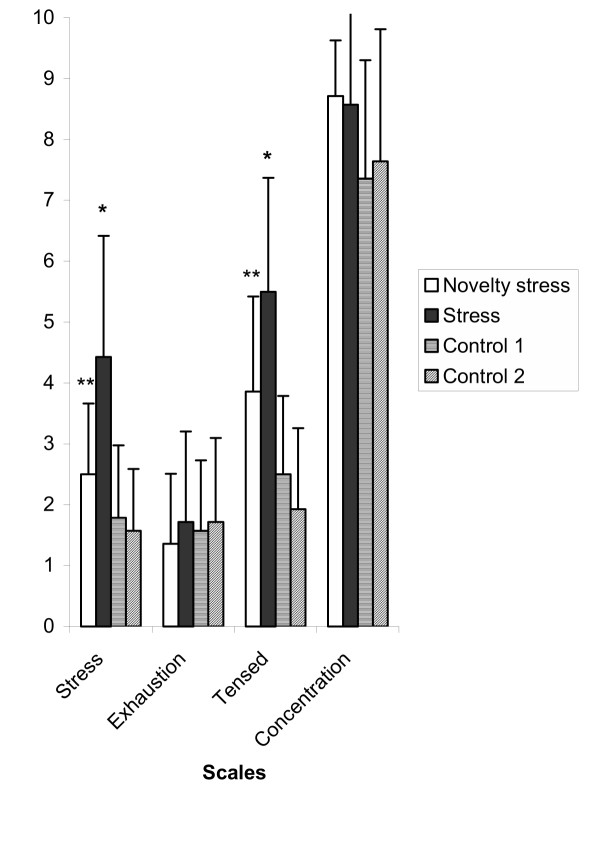
**Subjective experience of stress. **Self-reports on stress, tenseness, exhaustion and concentration. Data are presented as means ± SD (n = 14). * P < 0.005. Stress vs. novelty stress, control 1 and 2**P < 0.05. Novelty stress vs. control 1 and 2.

Self-reports on stress, tenseness, exhaustion, and concentration are presented in Figure 2. The participants experienced significantly more stress and tension in the stress period compared to the novelty stress, control 1 and 2 periods. Furthermore, the participants experienced significantly more tension in the novelty stress period compared to the control 1 and 2 periods. No differences were observed in the scales exhaustion and concentration between the periods.

### Physiological arousal (blood pressure and heart rate)

SBP, DBP, MAP and HR are presented in table 1. SBP, DBP and MAP were significantly increased in the novelty stress and stress periods compared to the resting periods. SBP, DBP and MAP were significantly higher in the novelty stress and stress periods (day 1) compared to control 1 and 2 (day 2), whereas HR tended to be higher in the novelty stress and stress periods compared to control 1 and 2 (P = 0.055). No differences were observed between the novelty stress and stress periods on day 1 and between the control 1 and 2 on day 2 for SBP, DBP, MAP and HR.

**Table 1:** Blood pressure and heart rate. Means (SD) of systolic blood pressure, diastolic blood pressure, mean arterial pressure and heart rate during the resting period, novelty stress and stress periods on day 1 and the resting period, control 1 and 2 periods on day 2 (n = 14).

	Systolic blood pressure (mmHg)	Diastolic blood pressure (mmHg)	Mean arterial Pressure (mmHg)	Heart rate (Beats*min^-1^)
Day 1				
Rest	132.3 (13.6)	84.0 (13.0)	102.4 (12.3)	78.7 (14.7)
Novelty Stress	142.0 (13.2)*	87.2 (13.1)*	107.8 (10.1)*	79.2 (15.5)
Stress	145.9 (12.0)*	91.3 (9.6)*	112.1 (9.0)*	80.80 (13.5)
Day 2				
Rest	121 (13.1)	77.8 (8.5)	94.7 (9.0)	74.2 (13.7)
Control 1	128.9 (13.0)	80.8 (7.8)	98.8 (8.4)	77.1 (13.0)
Control 2	130.2 (11.6)	82.7 (7.0)	100.2 (7.7)	75.1 (11.4)

* P < 0.005 Control 1 and 2 vs. novelty stress and stress.

**Figure 3:**
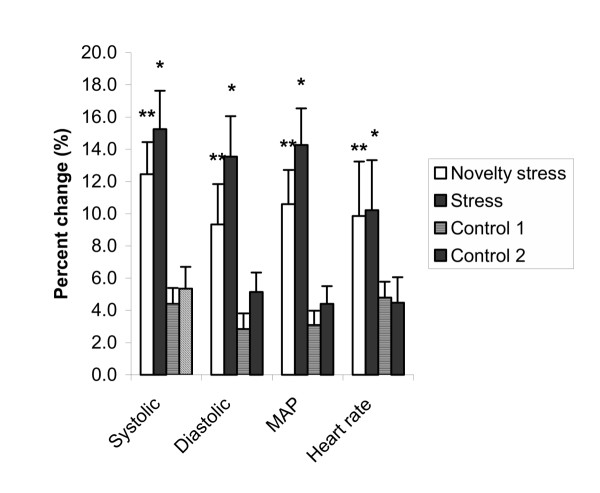
**Physiological arousal. **Percent change from the resting period for systolic blood pressure, diastolic blood pressure, mean arterial pressure and heart rate during the novelty stress and stress, and control 1 and 2 periods. Data are presented as means ± SE (n = 14). * P < 0.005 Stress vs. control 1 and 2. **P < 0.05 Novelty stress vs. control 1 and 2

When expressed as percent change from the resting period, the SBP, DBP, MAP and HR were significantly elevated in the novelty stress and stress periods compared to the control period. The SBP, DBP, MAP and HR increased with 15.2 ± 8.9%, 13.5 ± 9.4%, 14,3 ± 8.5% and 10.2 ± 11.6%, respectively in the stress period and with 12.4 ± 7.5%, 9.3 ± 9.4%, 10.6 ± 7.9% and 9.9 ± 12.6%, respectively, in the novelty stress period compared to the resting period (Figure 3).

### Position sense acuity (absolute error)

**Figure 4:**
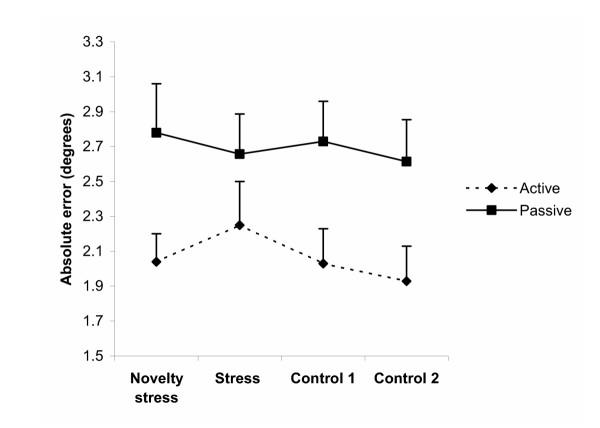
**Position sense acuity. **Passive and active position sense acuity. Absolute errors between target position and the reproduced position in the novelty stress, stress, control 1 and 2 periods. Data are presented as means ± SE (n = 14).

Figure 4 shows the novelty stress and stress, control 1 and 2 comparisons for AE. No significant changes in AE for passive position sense acuity and active position sense acuity were found when comparing the novelty stress, stress, control 1 and 2 periods. AE was significantly lower in the active procedure compared to the passive procedure. When testing the effect of the distances no differences in AE for 10°, 20° and 30° were found in the active and passive position sense tasks.

## Discussion

Significant changes in the cardiovascular parameters indicative of increased sympathetic activity during the electrical shock stressor and significant changes in self-reported tenseness and stress were demonstrated. However, the position sense acuity in the rotational plane of the lumbar spine was unaffected.

In view of the debate on a sympathetically mediated decrease in muscle spindle sensitivity the results provide limited information. The observation in the present study that an increase in sympathetic nervous activity, as indicated by the increase in blood pressure and heart rate, has no detrimental effect on position sense in the back has some important practical implications, but does not solve the problems on the potential role of the muscle spindle system. The maintained proprioceptive acuity may thus either reflect that a stress induced sympathetically mediated decrease in spindle sensitivity is of limited importance, or it could simply mean that a sympathetically mediated decree in spindles sensitivity does not occur in the present experimental conditions. Further, important to the hypothesis of the present study, that a state of high sympathetic activity would affect proprioception, is whether sympathetic nerves innervates muscle spindles and whether mental stress (in this study the response to the electrical shock stressor) increases muscle sympathetic activity to spinal muscles.

The hypothesis of stress induced disturbances of the sensitivity of the muscle spindle via sympathetic regulation originates from animal studies [18,19,32,33] demonstrating sympathetic fibres penetrating into the muscle spindle capsule [16] and the presence of adrenergic receptors inside the muscle spindle [17]. However, these findings may not be transferable into humans. Macefield et al. (2003)[20] failed to observe any changes in muscle spindle firing during a strong and sustained increase in MSNA in relaxed human leg muscles lending no support to the concept that the sympathetic nervous system can influence the sensitivity of human muscle spindles. This could explain the lack of effect of mental stress on position sense acuity in our study. Further, another study suggested that proprioception was unchanged or, in one condition, improved during muscle sympathetic activation lending partly support to the hypothesis of a sympathetic modulation of the muscle spindle [21]. Finally, Hjortskov et al.[34] demonstrated a facilitation of the short latency stretch reflex in the relaxed soleus muscle during manoeuvres known to increase MSNA, i.e. mental arithmetic, static handgrip exercise and post-handgrip ischemia. Although this is consistent with the idea that sympathetic nervous activity can exert a direct influence on human muscle spindles it is still unknown whether sympathetic nerves innervate human muscle spindles.

While it has consistently been shown that mental stress evokes an increase in MSNA e.g. [22,38], it is important to note the diversity of sympathetic outflow to different muscle groups i.e. during mental arithmetic an increased MSNA was seen in the lower limb but not in the upper limb [22]. Further, not all muscle spindles receive sympathetic innervations, and the proportion that does, varies between muscles [16]. Therefore, it may be that the low back muscles involved in the trunk movement in the present study, just as the arm muscles, are not under sympathetic control.

Further, if the low back muscles are under sympathetic control, the question arises as to whether MSNA was increased in the present study. In this regard, it has been shown that only high levels of mental stress increases the MSNA and that a decrease generally occurs during low levels of mental stress [35]. The effectiveness of the electrical shock stressor in activating the sympathetic system is assessed through its cardiovascular effects but it is unknown whether this stressor also elicits changes in MSNA and muscle spindle firing rate in the back muscles. However, arterial blood pressure responses have been proposed to reflect both cardiac and MSNA during mental stress. Compared to a study by Callister et al (1992) [38] reporting increases of 120–135% in MSNA, the cardiovascular changes were similar or even higher in our study. Likewise, compared to other studies using an electrical shock stressor [3,4,31], the physiological effects of the stressor were high. Therefore, if the low back muscles are under sympathetic control, it seems reasonable to assume an increased level of MSNA during the stress periods in the present study. It could however be that the level of MSNA during mental stress generally is not sufficient to influence the muscle spindle or that the low back muscles are not under sympathetic control as indicated above.

The type of proprioception tests may also explain the lack of change in position sense acuity in the present study. It has been suggested that primary muscle spindle afferents provide relatively more information on limb velocity whereas secondary muscle spindle afferents contribute mainly to limb position sense [15]. Interestingly, mental computation increased the response of the primary muscle spindle afferents while the secondary muscle spindle afferents exhibited no change in their sensitivity to stretch during mental computation [36,37]. Accordingly, it may be that proprioception testing designed to involve the primary muscle spindle afferents to a higher degree, as in replication of limb movement velocity, would be affected by mental stress.

Mental stress has been found to increase spine loadings during standardized lifting tasks [5,6,38] and to impair fine motor control [3,4]. Different mechanisms have been proposed. While Noteboom et al (2001a)[4] hypothesized elevated neuroendocrine activity during heightened arousal being responsible for the changes in motor performance, Davis et al (2002) [6] and Marras et al (2000) [5] suggested a biomechanical pathway leading to an overreaction of the musculoskeletal system i.e. in less controlled trunk movements and increases in trunk muscle coactivation. Further, it has been suggested that stress induced enhanced muscle sympathetic outflow to the muscle spindles may detrimentally affect motor performance and possible cause inefficient muscle use [18]. Contrary to that, Rossi-Durand (2002) [36] demonstrated that mental computation increased the muscle spindle sensitivity and suggested on that background that the increase in muscle spindle sensitivity could prepare the spindles to better play their role in proprioceptive information. The present study neither confirms nor disproves this suggestion.

A limitation of the study is the relatively low number of subjects participating in the study and that the order of the stress and control periods was not randomised. A larger study group may have influenced the results. Further, it could be argued that the short-term exposure to the stressor may have minimised the effects. However, despite the short-term exposure the cardiovascular stress indicators were markedly heighten during the "novelty stress" and the "stress" periods.

## Conclusion

Participants presented with the shock stressor experienced significant changes in cognitive and physiological measures of stress. However, the position sense acuity in the rotational plane of the lumbar spine was unaffected during the electrical shock stressor. Further human studies on different muscle groups and with different testing procedures are needed to clarify the effect of mental stress on proprioception and motor control.

## Competing interests

The author(s) declare that they have no competing interests.

## Authors' contributions

NH, CHK and NF formulated the study idea, the choice of techniques and the experimental design and analyzed the experimental results in collaboration. NH and CHK performed the experiments. NH, CHK and NF processed the data, performed the statistics and drew figures/tables. NH wrote the draft of the manuscript, which was revised in collaboration and agreed upon in its final version by all authors.

## Pre-publication history

The pre-publication history for this paper can be accessed here:


